# L'hémangiopéricytome méningé: une tumeur intracrânienne rare - à propos d'un cas

**DOI:** 10.11604/pamj.2014.17.223.2920

**Published:** 2014-03-21

**Authors:** Amal Errachdi, Amal Asabbane, Brice Nkoua Epala, Mariem Hemmich, Naoual Kabbali, Adama Diakité, Tayeb Kebdani, Noureddine Benjaafar

**Affiliations:** 1 Pôle de Radiothérapie, Institut National d'Oncologie, CHU Ibn Sina, Rabat, Maroc

**Keywords:** Hémangiopéricytome méningé intracrânial, chirurgie, radiothérapie, récidive, métastase, Intracranial meningeal hemangiopericytoma, surgery, radiotherapy, metastasis, métastase

## Abstract

L'hémangiopéricytomeméningé intracrânien est une tumeur rare qui représente moins de 1% des tumeurs cérébrales. Son aspect radiologique peut être trompeur et faire porter à tort le diagnostic de méningiome. Le diagnostic de confirmation est histologique. Le traitement repose sur la chirurgie et la radiothérapie. L’évolution après traitement est caractérisée par la fréquence des récidives et des métastases à distance, imposant un suivi prolongé. Nous rapportons l'observation d'un patient présentant un hémangiopéricytome intracrânien dont l’évolution était marquée par l'absence de récidive à 4 ans de suivi.

## Introduction

L′hémangiopéricytome (HP) est une tumeur mésenchymateuse rare, issue des péricytes de Zimmerman. Son aspect en tomodensitométrie (TDM) ou en imagerie par résonance magnétique (IRM) peut être trompeur et faire porter à tort le diagnostic de méningiome. L′HP est caractérisé par son potentiel malin et son taux élevé de récidives et de métastases à distance, justifiant une exérèse chirurgicale large et une radiothérapie complémentaire. Nous rapportons un cas d'HP intracrânien avec revue de la littérature.

## Patient et observation

**Figure 1:**
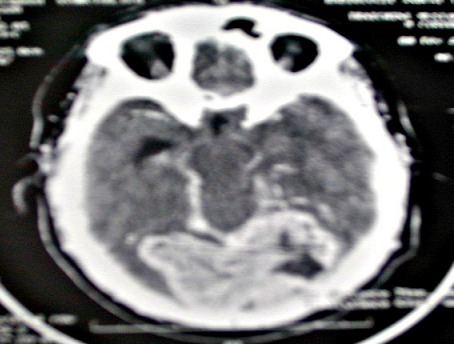
TDM cérébrale en coupe axiale de notre patient objectivant un rehaussement massif de l'hémangiopéricytome après injection du produit de contraste

**Figure 2:**
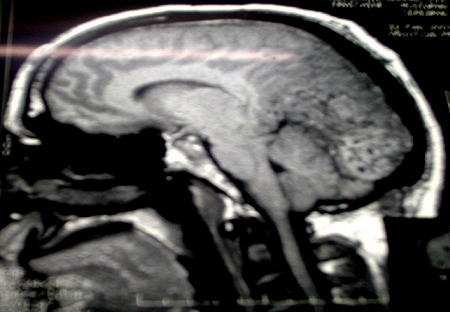
IRM cérébrale en coupe sagittale du patient montrant la localisation occipitale de la tumeur

**Figure 3:**
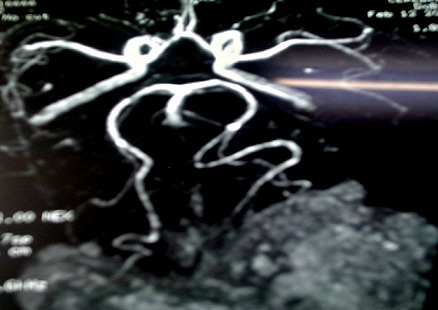
Artériographie cérébrale du patient montrant une tumeur hypervasculaire alimentée par les deux artères carotides

M. ET, âgé de 49 ans, avait consulté pour des céphalées intenses évoluant depuis 5 mois, associées àdes troubles visuels. L'examen avait objectivé une baisse bilatérale de l'acuité visuelle et un léger élargissement du polygone de sustentation à la marche, sans déficit neurologique. LaTDM cérébrale mettait en évidence un processus tumoral discrètement dense, se rehaussant massivement de façon hétérogène par le produit de contraste, mesurant 70×60mm de diamètre au niveau du cervelet de part et d'autre de la ligne médiane (Figure 1), étendu au lobe occipital gauche avec une discrète érosion osseuse de la table interne de l'os occipital et effet de masse sur la ligne médiane. A l'IRM cérébrale, il s'agissait d'une formation tumorale intéressant les lobes occipitaux des deux côtés de la ligne médiane prédominant à gauche (Figure 2), prenant le contraste de façon homogène et venant au contact de la partie inférieure du sinus longitudinal supérieur et des sinus latéraux montrant une importante hypervascularisation intratumorale. L'artériographie cérébrale avait décrit une tumeur hypervasculaire essentiellement alimentée par des branches des deux carotides externes (Figure 3). L'aspect radiologique évoquait un méningiome atypique hypervasculaire ou un hémangiopéricytome.

La biopsie avec étude immunohistochimique a permis la confirmation du diagnostic d'HP par la forte positivité du CD34. Les autres marqueurs EMA (l'antigène membranaire épithélial) et le GFAP (Glial fibrillary acidic protein) étaient négatifs.

Le patient avait refusé la chirurgie. Il a bénéficié d'une radiothérapie exclusive à la dose de 60Gy en deux séries au Cobalt 1. 25 MeV. Une première série de 40Gy en 20 fractions a été délivrée sur la tumeur avec une marge de 2cm par 2 champs latéraux droit et gauche suivie d'un complément de 20Gy en 10 fractions avec réduction des marges de 0,5cm. L’étalement total était de 43 jours. L’évolution a été marquée par l'amélioration de la symptomatologie clinique. Un scanner cérébral de contrôle fait après un an avait montré une régression tumorale estimée à plus de 70%. Le patient est resté en bon contrôle loco-régional et à distance avec un recul de 4ans.

## Discussion

L'HP est une tumeur vasculaire rare qui représente moins de 1% de toutes les tumeurs intracrâniennes. Il survient dans 56 à 75% entre 38 et 42 ans et atteint le plus souvent l’étage sus-tentoriel [1], ce qui était le cas de notre patient.

Initialement classés comme sous-types de méningiome par Bayley et al. [2], appelés « méningiomesangioblastiques » ou « méningiomes hémangiopéricytiques ». Cette classification fut controversée étant donnée l'origine différente de ces deux tumeurs: les HP proviennent des péricytes des capillaires qui entourent les méninges, alors que les méningiomes se développent à partir des cellules arachnoïdiennes [3]. Ce n'est qu'en 1942 que le terme d'HP a été introduit par Stout et Murray [4], qui publièrent les neuf premiers cas d'HP de la littérature. Depuis 1993, l′Organisation Mondiale de la Santé (OMS) classe l′hémangiopéricytome dans le groupe des « tumeurs méningées mésenchymateuses malignes non méningothéliales ». Sur le plan clinique, La symptomatologie initiale varie en fonction de la localisation et de la taille de la tumeur. L'atteinte supratentorielle se traduit principalement par des céphalées, et moins fréquemment par un déficit sensitivomoteur, des troubles visuels ou des crises comitiales. Les tumeurs de la fosse postérieure sont surtout responsables de troubles de l’équilibre, ce qui était le cas de notre patient. L'atteinte parasellaire est évoquée devant un syndrome de la loge caverneuse [3, 5]. L'imagerie cérébrale (TDM ou IRM)permet de décrire l'aspect et les caractéristiques de la tumeur faisant évoquer un HP sans toutefois pouvoir éliminer le diagnostic différentiel de méningiome. L'angiographie semble intéressante vue la nature hypervascularisé de l'HP [6]. L'immuno-histochimie permet de confirmer le diagnostic. Les HP n'expriment que des marqueurs mésenchymateux. Les cellules tumorales sont marquées par les anticorps dirigés contre le CD 34, mais sont négatives pour les anticorps anti-facteur VIII et la protéine S-100 [7].

Le traitement repose sur une résection chirurgicale suivie d′une radiothérapie. L′exérèse chirurgicale doit être aussi large que possible. Toutefois, la nature très hémorragique de la tumeur peut poser des problèmes majeurs d′hémostase et rendre l′exérèse délicate. Afin de réduire la vascularisation tumorale, une embolisation préopératoire est parfois réalisée avec des résultats variables selon les séries [5, 8].

Le rôle de la radiothérapie est actuellement unanimement admis: elle prolonge la période derémission, réduit le taux des récidives et augmente par conséquent la durée de survie [9]. La réponse à la radiothérapie est dose-dépendante: seules des doses supérieures à 50 Gy sont associées à une réduction statistiquement significative du taux de récidive locale [4, 9]. Cette radiothérapie doit être instaurée d'emblée, même si l'exérèse tumorale est jugée complète. Jeong H et al. [10] rapportent des taux de rémission à 5 ans de 100% chez des patients ayant eu une radiothérapie après la chirurgie et de 70, 3% chez les patients ayant eu une chirurgie seule. Pour Guthrie et al. [5], la radiothérapie après la première intervention prolonge la période de rémission de 34 à 75 mois et prolonge la survie de 62 à 92 mois. Dufour et al. [3] rapportent des taux de récidive de 12% et de mortalité nul dans le groupe de patients irradiés alors que dans le groupe de patients non irradiés les taux de récidive et de mortalité étaient respectivement de 88% et de 55%. L’évolution des HP est marquée par la fréquence des récidives locales et à distance. Le taux de rechutes locales varie selon les séries de 26 à 86% et dépend essentiellement de la qualité de l′exérèse et de la pratique d′une radiothérapie postopératoire [3, 5, 10].

L′extension à distance peut se faire le long du névraxe, par voie liquidienne ou par voie hématogène [3]. Les sites métastatiques les plus fréquents sont l′os, le poumon et le foie [3, 6]. La probabilité de survenue de métastases augmente avec le temps, 13% à 5 ans, 33% à 10 ans, pour atteindre 64% à 15 ans [3, 5, 10].

## Conclusion

L′hémangiopéricytome est une tumeur rare, de diagnostic difficile, souvent pris à tort pour un méningiome. Cependant, il est important d′évoquer le diagnostic en préopératoire, compte tenu des risques hémorragiques lors de l′intervention. L′imagerie est peu spécifique. La certitude diagnostique reste histologique, principalement basée sur l’étude immunocytochimique. Le traitement repose sur la chirurgie suivie de la radiothérapie qui permet de diminuer significativement les taux de récidives. Un suivi prolongé de ces patients est nécessaire, compte tenu de la fréquence des récidives et de la survenue parfois tardive de métastases.
